# Treatment of in-transit Merkel cell carcinoma by isolated limb infusion with cytotoxic drugs

**DOI:** 10.1093/jscr/rjac172

**Published:** 2022-04-12

**Authors:** Jake J Hindmarch, David J Coker, Richard Waugh, Peter C A Kam, John F Thompson, Robyn P M Saw

**Affiliations:** Melanoma Institute Australia, The University of Sydney, North Sydney, New South Wales, Australia; Department of Melanoma and Surgical Oncology, Royal Prince Alfred Hospital, Camperdown, New South Wales, Australia; Melanoma Institute Australia, The University of Sydney, North Sydney, New South Wales, Australia; Department of Melanoma and Surgical Oncology, Royal Prince Alfred Hospital, Camperdown, New South Wales, Australia; Faculty of Medicine and Health, The University of Sydney, Sydney, New South Wales, Australia; Department of Radiology, Royal Prince Alfred Hospital, Camperdown, New South Wales, Australia; Faculty of Medicine and Health, The University of Sydney, Sydney, New South Wales, Australia; Department of Anaesthetics, Royal Prince Alfred Hospital, Camperdown, New South Wales, Australia; Melanoma Institute Australia, The University of Sydney, North Sydney, New South Wales, Australia; Department of Melanoma and Surgical Oncology, Royal Prince Alfred Hospital, Camperdown, New South Wales, Australia; Faculty of Medicine and Health, The University of Sydney, Sydney, New South Wales, Australia; Melanoma Institute Australia, The University of Sydney, North Sydney, New South Wales, Australia; Department of Melanoma and Surgical Oncology, Royal Prince Alfred Hospital, Camperdown, New South Wales, Australia; Faculty of Medicine and Health, The University of Sydney, Sydney, New South Wales, Australia

## Abstract

Merkel cell carcinoma of the skin is a rare but aggressive malignancy, which predominantly affects older adults with fair skin. Isolated limb infusion (ILI) using melphalan and actinomycin D was first developed as a minimally invasive treatment option to treat unresectable metastatic melanoma confined to the limb. We report on a 62-year-old male with in-transit metastases (ITMs) treated with ILI to highlight the ongoing role this treatment has when all other therapies have been exhausted. At presentation, the patient had widespread ITMs in the right leg. Positron emission tomography scan demonstrated recurrent disease in the thigh and pelvis, and it was decided to treat the patient with ILI. The patient progressed well in the immediate post-operative period. The patient was able to mobilize from Day 6 post-ILI and was discharged on Day 10. There was an immediate clinical response seen in the lesions, with necrosis developing in the larger lesions.

## INTRODUCTION

Cutaneous Merkel cell carcinoma (MCC) of the skin is a rare but aggressive malignancy, which predominantly affects older adults with a fair complexion [1]. Other risk factors for MCC include male sex [2] and immunosuppression [3]. While the pathogenesis of MCC is probably multifactorial, there appear to be distinct pathways leading to its development, with Merkel cell polyomavirus-associated lesions predominating in the northern hemisphere, whereas ultraviolet radiation exposure appears to be the dominant risk factor in the southern hemisphere [4, 5]. In keeping with sun exposure as a risk factor, the most frequent anatomical locations for the primary lesions are the head and neck (43%), upper limbs and shoulders (24%), lower limbs and hips (15%) [6]. At the time of presentation, 26% of patients with MCC have regional lymph node involvement and 8% of patients have distant metastases [6]. The extent of disease at presentation is an important prognostic factor for survival.

Isolated limb infusion (ILI) using melphalan and actinomycin D was first developed as a minimally invasive treatment option for unresectable metastatic melanoma confined to a limb [7]. The use of ILI for other tumour types, including MCC, has been reported previously [8]. Advances in immunotherapy have reduced the need for ILI to control loco-regional disease. We report the following case to highlight the role of ILI in MCC following the failure of systemic therapy to control extensive and debilitating local disease.

## CASE REPORT

A 62-year-old male was diagnosed with MCC on his right shin in July 2018. He was treated by wide local excision and skin grafting, followed by adjuvant radiotherapy. He subsequently developed in-transit metastases (ITMs) and was treated with avelumab immunotherapy. Initially, there was a good response, but in early 2020, progressive nodal disease was noted involving inguinal and iliac lymph nodes. A groin dissection was performed, which was followed by six cycles of carboplatin and etoposide. New ITMs developed while he was on chemotherapy, and further radiotherapy was given to the right leg. After an initial response, the patient developed yet more ITMs and topical diphencyprone was trialled without success.

**Figure 1:**
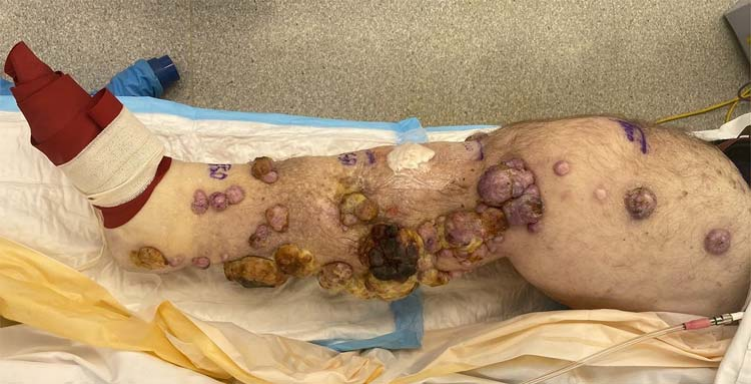
Right leg at commencement of ILI, with foot excluded using an Esmarch bandage.

At presentation, the patient had widespread ITMs in the right leg and thigh, which soaked through daily dressings and significantly impaired his quality of life (Fig. 1). A positron emission tomography scan also demonstrated recurrent disease in the proximal thigh and pelvis; however, given the exhaustion of other treatment modalities, significant impact on his quality of life and after discussion at a multidisciplinary meeting, the patient agreed to an ILI. His relevant co-morbidities included Type 2 diabetes mellitus, obstructive sleep apnoea, asthma and hypertension.

**Figure 2:**
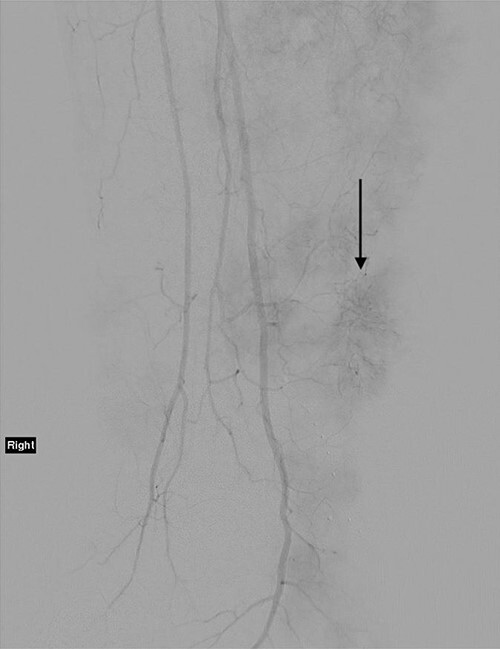
Angiogram of the right leg, showing the significant neoangiogenesis present in the large tumour masses; the arrow indicates one of the highly vascular tumour deposits.

The technique of ILI has been described previously. It is a minimally invasive procedure that involves isolating the blood supply of a limb in order to control the delivery of chemotherapy drugs into it and to avoid systemic drug toxicity. A tourniquet is applied to the proximal limb to quarantine the limb blood supply after the arterial and venous catheters have been placed to circulate high doses of chemotherapy throughout the limb for 30 minutes [9]. Figure 2 demonstrates a post-catheter placement digital subtraction angiography image of the diseased limb, showing the significant vascularity of the lesions that makes ILI an effective treatment by delivering cytotoxic chemotherapy in high doses directly to tumour deposits. The patient progressed well in the immediate post-operative period.

**Figure 3:**
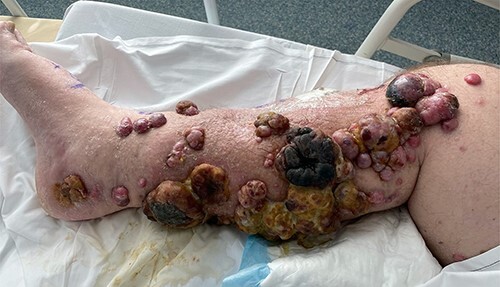
Right leg on Day 9 post-ILI, showing early changes of necrosis particularly in the larger tumour masses.

On Day 3 post-ILI, he was commenced on IV dexamethasone because of a threatened compartment syndrome, with increased pain on dorsiflexion of his ankle. His clinical condition improved, his serum creatinine kinase peaked at 1742 U/l the following day and continued to decline in keeping with his clinical progress. He was able to mobilize from Day 6 and was discharged on Day 10 on a weaning dose of steroids. There was an immediate clinical response seen in the lesions of the right leg, with necrosis developing in the larger lesions within 24 hours (Fig. 3).

Three weeks post-operatively, the patient re-presented with significant pain in the proximal right thigh and pelvis. Imaging revealed significant progression of the disease proximal to the tourniquet placed at ILI and excluded a deep vein thrombosis. The distal leg lesions had continued to necrose and involute. The palliative care team assisted in providing analgesia and symptom control.

## DISCUSSION

Despite the unsatisfactory ultimate outcome in this case, it illustrates that there may be a role for ILI in patients with MCC who have a significant burden of ITMs when other treatment modalities have failed to achieve disease control. Very careful patient selection is essential, and discussion of each patient’s remaining treatment options by a multidisciplinary team is important.

## CONFLICT OF INTEREST STATEMENT

J.F.T. has received honoraria for advisory board participation from BMS Australia, MSD Australia, GSK and Provectus Inc; travel support from GSK and Provectus Inc and support for conference attendance from Novartis. R.P.M.S. has received honoraria for advisory board participation from MSD, Novartis and Qbiotics and speaking honoraria from BMS and Novartis. The other authors have no conflict of interest to declare.

## FUNDING

This article has no funding source.

## PRIOR PRESENTATION

This case report has been previously presented at the Melanoma Institute Australia conference in 2021. This case report has not been published in any other journal.
